# Introduction—First Billion Years: Habitability

**DOI:** 10.1089/ast.2020.2314

**Published:** 2021-08-16

**Authors:** Edgard G. Rivera-Valentín, Justin Filiberto, Kennda L. Lynch, Irena Mamajanov, Timothy W. Lyons, Mitch Schulte, Abel Méndez

**Affiliations:** ^1^ Lunar and Planetary Institute, Universities Space Research Association, Houston, Texas, USA.; ^2^ Earth-Life Science Institute, Tokyo Institute of Technology, Tokyo, Japan.; ^3^ Department of Earth and Planetary Sciences, University of California Riverside, Riverside, California, USA.; ^4^ Planetary Science Division, NASA Headquarters, Washington, District of Columbia, USA.; ^5^ Planetary Habitability Laboratory, University of Puerto Rico Arecibo, Arecibo, Puerto Rico.

**Keywords:** Habitability, Origins of life, Biosignatures, Exoplanets, Analog studies

## Abstract

The physical processes active during the first billion years (FBY) of Earth's history, such as accretion, differentiation, and impact cratering, provide constraints on the initial conditions that were conducive to the formation and establishment of life on Earth. This motivated the Lunar and Planetary Institute's FBY topical initiative, which was a four-part conference series intended to look at each of these physical processes to study the basic structure and composition of our Solar System that was set during the FBY. The FBY Habitability conference, held in September 2019, was the last in this series and was intended to synthesize the initiative; specifically, to further our understanding of the origins of life, planetary and environmental habitability, and the search for life beyond Earth. The conference included discussions of planetary habitability and the potential emergence of life on bodies within our Solar System, as well as extrasolar systems by applying our knowledge of the Solar System's FBY, and in particular Earth's early history. To introduce this Special Collection, which resulted from work discussed at the conference, we provide a review of the main themes and a synopsis of the FBY Habitability conference.

## 1. Introduction

The First Billion Years (FBY) Habitability conference was the fourth and final installment of the Lunar and Planetary Institute's The FBY series, which summarized building and evolving planets from accretion to habitability. Continuing with the goal of the initiative, this conference was intended to foster multi- and interdisciplinary discussion on the processes and physical conditions that affected the development of potentially habitable environments during the FBY, how such environments evolved over time, and how such ideas may be tested with current and future laboratory measurements, field analog studies, spacecraft missions, and astronomical observations.

The focus of this conference was habitability and the processes that produced the conditions conducive to life and led to the emergence of life on Earth. In addition, the conference included discussions on the potential for the emergence of life on other Solar System bodies and extensions to extrasolar systems. There was a particular emphasis on integrating the diverse fields of study in astrobiology and a focus on the intersections of these fields as they apply to understanding the conditions that allow life to emerge and establish on a planet. Thematically, this FBY conference centered around the study of the origins of life, habitability, and the search for life beyond Earth. The discussion was principally guided by three key questions:

1. What prebiotic reactions were possible during the period when life emerged on Earth and, conversely, what environmental conditions were needed on early Earth to foster key prebiotic reactions?

The geologic record has established that life arose on Earth sometime during its FBY (Walter *et al.*, 1980; Pace, 1991; Cairns-Smith *et al.*, 1992; Westall *et al.*, 2001; Allwood *et al.*, 2007; Van Kranendonk *et al.*, 2008; Dodd *et al.*, 2017; Schopf *et al.*, 2018). The same record can also be used to define the environmental conditions and chemical building blocks that were present that either led to or potentially encouraged life to arise on Earth. In addition, studies of life's chemistry and the diversity of present-day life, including adaptations to extreme environments, provide a means to presently explore the origins of life. However, to refine our understanding of the chemical pathways that led to life on Earth, we must identify the key measurements needed to define the environmental and chemical parameter space.

2. Did the timing of habitable environments on Earth coincide with the timing of potentially habitable environments on other planetary bodies (*e.g.*, Mars, Venus, Europa), as well as the timing of global-scale processes, such as a Late Heavy Bombardment?

Planetary-scale events in our Solar System may have altered the pathway for the emergence and evolution of life on Earth and elsewhere. Indeed, environmental conditions on Earth and elsewhere drastically changed during the FBY, which not only impacts the establishment of life but also the potential for biosignature preservation and thus the search for life on other worlds.

3. What are the defining factors that influence the habitability of exoplanets through time and what future technologies are required to identify habitable worlds?

Constraints from the time when life emerged on Earth—the FBY—along with context from our own Solar System allow us to elucidate the conditions needed for planetary habitability beyond our Solar System. Conversely, the myriad of exoplanet detections provides important context for the evolution of our own Solar System. Indeed, the now numerous exoplanet detections are challenging our notions of the diversity of planets and their potential habitability. To detect habitable exoplanets, however, we must first understand the conditions and processes under which Earth and other solar system worlds became habitable, and develop the technology required to observe them both by spacecraft missions (within our Solar System) and astronomical observations (beyond).

To ensure that these topics were fully discussed at the conference, a scientific organizing committee of 15 was formed with emphasis on the diversity of subject matter expertise, gender, ethnicity/race, and geographic location. The 3-day conference, which was supported through the Lunar and Planetary Institute, was held in Big Sky, MT. The conference began with a field excursion to an analog site for conditions during the FBY of the Solar System: Yellowstone National Park. The field site was used to prompt discussion on the biologic potential of hydrothermal systems in volcanic settings and the similar potential habitability of impact-generated hydrothermal systems.

Each day of the meeting centered around one of the conference themes and was started by an invited speaker. The speaker introduced the topic and provided a state-of-the-field discussion such that all conference attendees, regardless of expertise, were provided with the appropriate background to participate in the day's discussions.

In this special collection, research and review articles resulting from the meeting are presented. Diverse topics are covered including revisiting what martian meteorite ALH 84001 constrains about the habitability of Early Mars (Treiman, 2021, this issue), an investigation into Earth's oxygenation history using banded iron formations (Schulz, 2021, this issue), and a new geobiology-driven nomenclature for astrobiological *in situ* observations and sample analyses (Perl *et al.*, 2021, this issue). In this report, we summarize the conference themes, the discussion at the workshop, and the questions still to be investigated about forming habitable planets and the potential emergence of life within our Solar System and beyond.

## 2. Thematic Overview

### 2.1. Origins of life

The studies of the origin of life began in the 1920s with the Oparin–Haldane theory (Haldane, 1929; Oparin, 1967), which suggested that the first organic compounds formed under a reducing atmosphere triggered by ultraviolet (UV) irradiation. The organic compounds would then rain down into the primeval ocean and aggregate to form coacervates, droplets formed upon liquid–liquid separation of immiscible phases and eventually evolve into cells. In 1952, Miller and Urey experimented with a setup mimicking Oparin–Haldane conditions to produce organic compounds, including several amino acids from gaseous ammonia, methane, hydrogen, and water vapor (Miller, 1953). The straightforward synthesis of adenine from hydrogen cyanide discovered by Oró (1961) expanded the inventory of prebiotic molecules to the precursors of nucleic acids.

In 1962, Rich (1962) proposed that RNA might be able to support both genetics (by being replicated) and phenotype (by having catalytic activity). This proposal gave rise to the RNA World theory, a hypothetical stage in the evolutionary history of life on Earth, in which RNA molecules proliferated before the advent of DNA or proteins (Neveu *et al.*, 2013). The discovery of ribozymes (Kruger *et al.*, 1982; Guerrier-Takada *et al.*, 1983) in the early 1980s has further reinforced the RNA World theory (Cech, 2012).

Alternative approaches, classified as Metabolism First theories, claim that metabolism might have preceded life and focus on studying catalytic chemical reaction systems. Such approaches include the study of carbon fixation driven by the reducing power of ferrous sulfide and hydrogen sulfide in conjunction with pyrite formation (Wachtershauser, 1988), by photocatalytic zinc sulfide minerals in a subaerial “Zinc World” scenario (Mulkidjanian, 2009), or by geochemical energy sources in the deep-sea hydrothermal vents (Russel and Hall, 1997; Martin *et al.*, 2008).

Many origins of life theories, including the nonexhaustive examples described above, focused on recreating certain modules of modern life under prebiotic conditions. Since life, as we know it, is now highly evolved, it is conceivable that early metabolism and replication were not based on the same chemical mechanisms as in modern life. The paradigm is akin to an example of technological development. A modern state-of-the-art television set differs considerably from its historic precursor based on cathode-ray technology. If one is given only a modern light emitting diode television and asked to figure out the origin of television technology without any historical information, the analysis is unlikely to trace the origin of television technology back to cathode-ray sets. In origin of life studies, there is a value in exploring bottom-up approaches focused on understanding the potential evolutionary pathways in prebiotic chemistry that are not necessarily inspired by modern biology.

Several experimental studies have attempted to replicate prebiotic conditions. The formation of biological building blocks has been reported in the abovementioned Miller-Urey experiment, under high pressure and temperature in geothermal vent conditions (Cody *et al.*, 2000), through hydrogen cyanide chemistry (Moser *et al.*, 1968), and in Fischer–Tropsch-type reactions (McCollom *et al.*, 1999). Furthermore, interesting “life-like properties,” such as functioning autocatalysis and self-assembling nucleoside analogues, have been discovered in the formose reaction (Socha *et al.*, 1980; Huskey and Epstein, 1989; Simonov *et al.*, 2007) and in cyanuric acid–triamino pyrimidine coupling (Cafferty *et al.*, 2013; Chen *et al.*, 2014), respectively.

Many of these prebiotic systems share a common characteristic—they are messy. The systems produce vast multicomponent mixtures of compounds through an abundance of reaction pathways. When messy prebiotic systems reach steady state or equilibrium, they produce heterogeneous intractable polymeric structures, dubbed “tar” or “asphalt” (Benner, 2014). Alternatively, the enzyme-controlled world of biochemistry is characterized by complex yet organized chemical networks with clearly defined reaction mechanisms and product diversity. The messy chemistry hypothesis conceptualizes the transition between the uncontrolled prebiotic chemistry and biochemistry through the utilization of methods of organization in chemistry and building a chemical system capable of evolution (Guttenberg *et al.*, 2017). Some examples of studies of the messy prebiotic chemistry are described below.

#### 2.1.1. Polyesters as a model system for the study of messy chemistry

An ester linkage or bond connects a carbon atom via a double bond to an oxygen atom and a single bond to an oxygen atom bearing the alkyl or aryl group. A peptide bond formed upon a condensation between a carboxylic acid and an amine differs from an ester linkage formed upon condensation between a carboxylic acid and an alcohol by one atom only (nitrogen instead of oxygen). While the formation of an ester linkage is more thermodynamically favorable than the formation of a peptide bond, unlike peptides, oligoesters cannot form a hydrogen bond along their backbones, limiting their folding abilities. Nevertheless, oligo- and polyesters have been considered prebiotic alternatives to peptides (Rich, 1971).

The formation of an ester linkage, though, is more thermodynamically favorable than the formation of a peptide bond. Weber (1989) showed that drying-down glyceric acid at 80°C leads to its condensation into polyesters with lengths of up to 25-mers. Polyesters were shown to form in dry–wet cycles at varying temperatures from malic acid. A dry-down of five α-hydroxy acids (αHAs) was shown to generate vast, likely sequence-complete libraries (Chandru *et al.*, 2018).

#### 2.1.2. Polyester protocell compartments

Compartmentalization was likely essential for primitive chemical systems during the emergence of life, both for concentration and leakage prevention of the essential components and providing the necessary microenvironments for the protometabolic processes (Monnard and Walde, 2015). Life as we know it uses lipid bilayer and protein-based compartments, but it is conceivable that primitive life used alternative, easier to assemble compartments. One example of such primitive compartments could have been the membrane-less microdroplets produced upon polyesterification of αHAs (Jia *et al.*, 2019). These compartments can segregate and compartmentalize fluorescent dyes and fluorescently tagged RNA. Furthermore, the protein function within the membrane-less microdroplets maintain their folded structure, while RNA function is preserved in the presence of microdroplets, suggesting the potential relevance of such droplets to various origins of life models.

#### 2.1.3. Protoenzymes

Enzymes are biological macromolecular catalysts. Enzymes are composed of an active site, a metal ion, cluster, or a small molecule augmented by globular protein or RNA. The function of the biopolymer scaffold is to specifically bind, orient within the active site, and encapsulate substrates in a suitable microenvironment. Highly functional proteins and RNA strands would not have been readily available prebiotically. It is conceivable that simple polymers substituted RNA or protein scaffold at the early stages of chemical evolution.

Hyperbranched polymers, intrinsically globular tree-like structures, are an attractive candidate to explore in this context as they can be straightforwardly synthesized from multifunctional monomers. Tertiary amine-bearing hyperbranched polyesters prepared in a simple dry-down reaction and used without purification were shown to form hydrophobic pockets as a reaction-promoting medium for the Kemp elimination reaction (Mamajanov and Cody, 2017). Hyperbranched polyethyleneimine and glycerol citrate polymer-supported ZnS nanocrystals were shown to possess significant stability and photocatalytic properties (Mamajanov *et al.*, 2020) making them an attractive model for the study of the origin of life under the “Zn world” theory (Mulkidjanian, 2009). “Zn World” suggests that life emerged within compartmentalized, photosynthesizing ZnS formations of hydrothermal origin, assembled in subaerial settings on the surface of the early Earth.

#### 2.1.4. Selectivity in messy systems

Selectivity is a central concept in chemical reactivity. Chemical reactions must be efficient and selective to be usable in synthesis; enzyme-controlled biochemical reactions are just significantly better fine-tuned toward small differences in reactant's structure and isotopic composition. Chemical syntheses routinely utilize selective mechanisms, including differential activation of specific chemical bonds, selection based on molecular topology, and particular catalyst deactivation (Balcells *et al.*, 2016).

Selectivity in prebiotic reactions is an area of active research. Ricardo *et al.* (2004) showed that borate minerals selectively stabilize sugars in the formose process, maximizing the sugar yields. Frenkel-Pinter *et al.* (2019) demonstrated that cationic amino acids found in modern peptides are more likely to incorporate into depsipeptides, polymers with mixed peptide and ester backbone, via ester-amine exchange process than nonbiological amino acids. In another example, polyesterification of citric acid and glycerol under wet–dry cycling conditions preferentially yielded more soluble hyperbranched, tree-like structures, over rigid insoluble crosslinked polymers, featuring multiple linkages between polymer chains (Mamajanov, 2019).

#### 2.1.5. Autocatalysis in messy systems

Chemical systems capable of templating and catalyzing their own production, or self-replicating systems, have been suggested to represent a link with the origin of life, and their behavior could perhaps provide a model for prebiotic chemical evolution. Autocatalytic sets, a self-sustaining chemical reaction network in which all the molecules mutually catalyze each other's formation from a basic food source, are believed to have played a role in the origin of life.

Various autocatalytic sets were studied using an artificial chemistry approach, a study of abstract chemical reaction networks, and emergent dynamical properties within the abstract networks (Banzhaf and Yamamoto, 2015). Several autocatalytic sets, although small and highly engineered, were constructed experimentally (Sievers and von Kiedrowski, 1994; Ashkenasy *et al.*, 2004; Lincoln and Joyce, 2009). Hordijk *et al.* (2019) argued that autocatalytic sets arise in a system with sufficient molecular diversity, suggesting exciting new avenues in the theoretical and experimental studies of messy chemistry.

#### 2.1.6. Information handling mechanisms in prebiotic chemistry

The concept of information handling is naturally built into the RNA theory, which proposes that RNA performed the information storage and transmission functions reserved for DNA in modern biology (Neveu *et al.*, 2013). The amyloid world theory proposes an alternative information handling mechanism (Maury, 2009, 2018). The theory focuses on the aggregation of short peptides into β-sheet amyloid conformers that in turn can self-propagate based on a templated self-replicative mechanism similar to that of prions. The theory further proposes that environmentally sensitive template-assisted replication cycles would generate a variety of amyloid polymorphs on which evolutive forces can act, and the fibrillar assemblies can serve as scaffolds for the amyloids themselves and for ribonucleotides, proteins, and lipids. In another peptide-based theory, Drexler (2018) proposed a mechanism of peptide replication through imprint-mediated templating for prebiotic peptide replication.

### 2.2. Habitability

Better understanding of the relationships among Earth's earliest environments, sustained habitability in those settings, and the conditions favorable to prebiotic chemistry and the beginning of life are exciting frontiers in origin research. Equally exciting is understanding our history of sustained habitability over billions of years in the face of dramatic change, such as a warming sun, cooling interior, growing and interacting continents, an evolving atmosphere, and life's initial contributions to its own environments.

The many diverse chapters of this history are spliced together by the common thread of sustained habitability—with persistent life over many if not most of the same chapters. How Earth maintained these conditions over billions of years in the face of so much change is teaching us important lessons in the exploration for life beyond our planet and Solar System, including the search for biosignature gases in the atmospheres around distant exoplanets (Schwieterman *et al.*, 2018). The contribution of Lyons *et al.* (2021, this issue) moved beyond life's beginnings to explore a long and vital period of latter life on Earth. By examining the parallel coevolution of the oceans and atmospheres, we learn more generally about essential drivers and feedbacks that helped make Earth habitable over most of its history—and may do the same on other, distant worlds.

The oldest signs of animal life appear in the geologic record 600 to 700 million years ago (Love *et al.*, 2009). For the 4 billion years prior, our planet experienced dramatic changes that paved the way for this milestone. Beyond the establishment of Earth's earliest oceans ∼4.3 billion years ago (Ga) (Mojzsis *et al.*, 2001; Wilde *et al.*, 2001), the single most important environmental transformation in history may have been the first permanent rise of atmospheric oxygen (O_2_) around 2.3 to 2.4 Ga [as reviewed in Lyons *et al.* (2014) and Catling and Zahnle (2020)]. Before this Great Oxidation Event (GOE), Earth's atmosphere and oceans were virtually devoid of O_2_, which is essential to all macroscopic life. Yet an O_2_ increase to anything close to modern levels was a long, drawn out, multistepped, and dynamic process. It is likely, for example, that there was a long delay between the first biological production and accumulation of O_2_ in the surface ocean, tentatively placed at ∼3.0 Ga (Planavsky *et al.*, 2014a; Satkoski *et al.*, 2015), and persistent, appreciable atmospheric accumulation recorded at the GOE.

Recent evidence suggests transient episodes of increased oxygenation before the GOE (Anbar *et al.*, 2007), and once permanently present in the atmosphere, O_2_ may have risen to very high levels before nose diving at ∼2.0 Ga (Bekker and Holland, 2012; Hodgskiss *et al.*, 2019; reviewed in Lyons *et al.*, 2014). At least a billion years of dominantly O_2_-free conditions in the deep ocean followed (Canfield, 1998; Scott *et al.*, 2008)—beneath an atmosphere and shallow ocean perhaps much leaner in O_2_ than previously thought (Lyons *et al.*, 2014; Daines *et al.*, 2017). Oxygen deficiencies and associated limitations in nutrients (Reinhard *et al.*, 2017; Laakso and Schrag, 2019; Ozaki *et al.*, 2019) may, in turn, have set a challenging course for many of the oceans' inhabitants (Crockford *et al.*, 2018)—including early complex life during its initial development and ecological expansion.

Recent evidence, though, also suggests the possibility of transient oxygenation events against this low baseline, perhaps coupled to emplacement of large igneous provinces that could have stimulated oxygenation and evolution through nutrient inputs associated with their weathering (Large *et al.*, 2019; Diamond *et al.*, 2020).

The latest data suggest that these billion-plus years of intermediate O_2_ were followed by an increase in ocean-atmosphere O_2_ contents and eukaryotic diversity 750 to 800 million years ago (a Neoproterozoic Oxidation Event) (Planavsky *et al.*, 2014b; Cohen and Macdonald, 2015), coincident with a time of eukaryotic innovation and their growing contributions to marine biomass as algal production (Brocks *et al.*, 2017; Zumberge *et al.*, 2020). Multiple geochemical and paleontological records point to a major biogeochemical transition at that time, but whether and how rising biospheric O_2_ triggered innovation in eukaryotic ecology, including the emergence of animals, are a matter of spirited debate.

The contribution by Lyons *et al.* (2021) in this issue focuses on the transition from the so-called boring billion to a more oxygenated world around 800 Ma—emphasizing diverse geochemical and paleontological records of concurrent environmental and biological change and the baseline conditions that preceded this jump. This comparison suggests temporal and perhaps mechanistic relationships between dynamic biospheric oxygenation and life, including the early rise of animals. Novel, rock-bound proxies and complementary numerical models are now steering our views of coevolving life and marine and atmospheric chemistry, including greenhouse gas controls on climate. New findings are revealing various states of planetary habitability that differ greatly from the Earth we know today. These “alternative Earths,” including our middle chapters, are helping to guide our search for life elsewhere in the Universe at a time of rapid discovery of exoplanets.

### 2.3. Search for life beyond Earth

In considering whether there is life elsewhere in the Solar System or the rest of the Universe, it is instructive to note that the only example we have so far of life is on the Earth. Understanding how life exists on the Earth (“life as we know it”) provides a great deal of information about the mechanisms, structures, energy sources, and environments we might expect for life beyond Earth. Life on Earth is very adaptable, but throughout its history has been and is dominated by microscopic, single-celled forms of life. It needs liquid water, a source of external chemical energy, and access to the elements necessary to build cellular structures and generate the machinery that allows it to extract energy from the environment and reproduce. One of the features of the environment that defines habitability is geochemistry; it provides the elements in bioavailable forms and contains the chemical disequilibrium necessary to provide energy sources for metabolism. In almost every environment where liquid water exists on Earth, within certain limits, life exists.

Life in what are considered to be extreme environments on Earth demonstrates the myriad ways microorganisms are able to take advantage of a great variety of energy sources and to thrive under conditions once thought to be uninhabitable. We can take lessons from these remarkable forms of life and use them as a guide to understanding the potential for life beyond Earth. Genetic sequences of a common, highly conserved ribosome point to a common ancestor (or more likely a community of ancestors) that lived at elevated temperatures and were chemotrophic and autotrophic (Woese *et al.*, 1990; Stetter, 1996; Pace, 1997; Boussau *et al.*, 2008; Braakman and Smith, 2014; Forterre, 2015). These factors all point to searches for life beyond Earth that should include the prospect of only single-celled organisms (microorganisms), evaluation of the geochemistry, and understanding that chemical reactions among liquid water and rock may provide the metabolic energy sources for life, rather than photosynthesis (although the latter obviously cannot be ruled out).

Evidence for life in the rock record on Earth takes a variety of forms and points to fairly sophisticated forms of life well established by as early as 3.5 billion years ago (Walter *et al.*, 1980; Westall *et al.*, 2001; Allwood *et al.*, 2007; Van Kranendonk *et al.*, 2008; Dodd *et al.*, 2017; Schopf *et al.*, 2018) and perhaps older (from rocks that are 3.8 Ga in Greenland). Most of the evidence is in the form of lithified microbial mats known as stromatolites, although there are chemical and isotopic signatures in stromatolites and other rocks that are interpreted to result from biological activity as well (Schidlowski, 1988).

One of NASA's strategic objectives in planetary science is to “ascertain the content, origin, and evolution of the Solar System and the potential for life elsewhere” (NASA, 2018). Much of our scientific research and many of our spacecraft are geared toward understanding the conditions that allow life to emerge and persist in the Solar System. Of special interest as possible locations where life may exist beyond Earth (or may have existed in the past) are Mars and icy satellites of the giant planets Jupiter and Saturn, such as Europa and Enceladus, which have liquid water oceans beneath icy crusts. NASA is currently developing missions to Mars and Europa (one of the moons of Jupiter), with the goal of seeking signs of life. The Mars 2020 rover, Perseverance, will seek signs of ancient life on the martian surface with a suite of instruments designed for detecting potential biosignatures, as well as cache samples for possible future return to Earth (Williford, 2018; iMOST *et al.*, 2019).

## 3. Workshop Summary

As stated above, Earth is our one data point for life, and therefore, the focus for studies of making habitable environments and the origin of life. Presentations at the workshop focused on using the terrestrial rock record to constrain habitable environments and their applicability to Mars, ocean worlds, and planetary bodies beyond our Solar System. This was initiated by discussions during the field trip. Here we summarize the key points of the Yellowstone analog site, other analog sites presented at the workshop, the applicability of this research to exoplanets, and finally, important open questions that still need to be addressed for making habitable planetary bodies, understanding the emergence of life on Earth, and how to search for life on other bodies.

### 3.1. Field trip to Yellowstone National Park sites

#### 3.1.1. Hydrothermal systems: why do we care about them in the FBY?

Hydrothermal systems are key habitability and origin of life environments because they provide heat, redox gradients, and a diversity of raw materials that are valuable for both early life and prebiotic chemistry (Des Marais and Walter, 2019). The interaction between the heated fluid and rocky material creates chemical disequilibria that both ancient life and modern life have been able to take advantage on Earth (Djokic *et al.*, 2017; Colman *et al.*, 2019; Dick, 2019). These dynamic environments are also thought to be ideal platforms for prebiotic chemistry and the origin of life (Barge *et al.*, 2019; Damer and Deamer, 2019). The unique geochemical nature of hydrothermal systems also provides for excellent biosignature preservation (Hays *et al.*, 2017). Furthermore, they are seemingly common across our Solar System, increasing their significance in the potential origin and maintenance of life on other planets in our Solar System (Sparks *et al.*, 2017; Waite *et al.*, 2017; Barge, 2018; Ruff *et al.*, 2019; Brown *et al.*, 2020; Castillo-Rogez *et al.*, 2020).

There are three major sources of hydrothermal systems on planetary bodies: impact-induced, cryovolcanism-indicated, and volcanism-induced. Impact-induced hydrothermal systems are the result of hypervelocity impact events capable of forming morphologically complex craters (*e.g.*, >2–4 and >5–10 km on Earth and Mars, respectively) into H_2_O-rich solid planetary surfaces (Osinski *et al.*, 2013). The shock pressures and heat generated by these impact events result in melted and/or heated rock material interacting with water to create and sustain hydrothermal systems. Depending on the characteristics of the system, they can survive for as little as 1500 years or as long as ∼2.3 million years (Jõeleht *et al.*, 2005; Kring *et al.*, 2020). On Earth there are ∼70 (out of the 180 total on the planet) known impact craters with evidence of hydrothermal activity and there are numerous studies of putative impact-generated systems on Mars and other potentially habitable worlds in our Solar System (Osinski *et al.*, 2013; Carrozzo *et al.*, 2017; Bowling *et al.*, 2019; Schenk *et al.*, 2019; Singh *et al.*, 2019).

Cryovolcanism can be an indicator of potentially present hydrothermal systems on icy worlds (Neveu *et al.*, 2015). While impact-generated hydrothermal systems are indeed possible on the surface of icy worlds, it is mostly thought that icy world hydrothermal systems likely exist/existed due to tidal or radiogenic heating of mantle material (Waite *et al.*, 2017; Bowling *et al.*, 2019).

Volcanism-driven hydrothermal activity occurs when silicate magma (either extrusive as lava flows or intrusive as dikes, sills, and plutons) interacts with, and heats, the crust to mobilize any fluids present (as liquid water, brine, or ice within the pore space of the country rock, ice within a cryosphere, or bound in hydrated minerals). This interaction would produce contact metamorphism with the country rock and generate a hydrothermal system with any fluids present (Semprich *et al.*, 2019; Griffiths, 2000; Hochstein and Browne, 2000; Costello *et al.*, 2020). The timescale and lateral extent of such systems depend on the temperature cooling rate of the magmatic body, the physical nature of the country rock sediments the lava contacts—specifically, whether the material is consolidated versus unconsolidated and its porosity—and any recharge to the fluids (Griffiths, 2000).

Such hydrothermal systems represent habitable environments and may even be where life began on Earth (Farmer, 1996; Shock, 1996; Shock *et al.*, 1998; Nisbet and Sleep, 2001; Pirajno and Van Kranendonk, 2005).

Due to this potential for habitability, investigations of hydrothermal activity have been a top priority for exploration of the martian crust (Schulze-Makuch *et al.*, 2007). On Mars we see a growing body of evidence of past hydrothermal features in numerous places, including the Gusev Crater, Arabia Terra, and even the Mars, 2020 landing site: Jezero Crater (Allen and Oehler, 2008; Schmidt *et al.*, 2008; Ruff *et al.*, 2011; Filiberto and Schwenzer, 2013; Arvidson *et al.*, 2014; Turner *et al.*, 2016; Carrozzo *et al.*, 2017; Ruff *et al.*, 2019; Singh *et al.*, 2019; Tarnas *et al.*, 2019; Brown *et al.*, 2020). We also see a growing body of evidence for the possibility of hydrothermal systems on icy/ocean worlds in our Solar System (Neveu *et al.*, 2015; Sparks *et al.*, 2017; Waite *et al.*, 2017; Bowling *et al.*, 2019; Schenk *et al.*, 2019; Castillo-Rogez *et al.*, 2020).

The diversity of terrestrial hydrothermal environments provides excellent opportunities for analog studies that can provide insights into the potential chemical conditions on other worlds. Furthermore, studies of hydrothermal ecosystems, as well as the chemical reactions facilitated in these environments, can provide clues to the potential habitability of worlds such as Mars and Enceladus. For example, hydrothermal vents have commonly been used as analog environments for icy/ocean worlds (Russell *et al.*, 2014). Of course, the hydrothermal features in Yellowstone National Park serve as analogues for multiple aspects of life on other planets. We study the geomorphology of hot springs, geysers, and mud pots, to learn how to identify them on other planetary bodies. We study processes in Yellowstone to understand how microbes and biosignatures could be preserved within hot spring deposits, fumaroles, and mud volcanoes. Finally, we study the microbes living in the extreme environments of Yellowstone to understand the tools and strategies that microorganisms use to survive in diverse hydrothermal environments. For all these reasons, the Yellowstone National Park has served as a seminal ecosystem for rising astrobiologists to learn about the science of astrobiology analog environments.

#### 3.1.2. The diverse ecosystems of Yellowstone National Park: a potential window into early life in the Solar System

The Yellowstone National Park is one of the largest and most studied hydrothermal systems on Earth. Contained within the park's boundaries are more than 10,000 hydrothermal features, including the largest concentrations of geysers, springs, mud pots, and fumaroles, with a diversity of geochemical conditions, including pH ranging from 1.5 to 10, gases from magmatic/mantle, crustal, and meteoric sources, and surface deposits ranging from silica sinter to native sulfur (Hurwitz and Lowenstern, 2014; Gonsior *et al.*, 2018).

In addition to the geophysical and geochemical diversity in Yellowstone, there is an incredible amount of microbial diversity living throughout these hydrothermal features. The microbes living in these varying extremes help us learn more about how life could arise, survive, and leave preserved biosignatures on early Earth and other habitable worlds (Spear *et al.*, 2005; Osburn *et al.*, 2011; Inskeep *et al.*, 2013; Hays *et al.*, 2017). For example, the Norris Geyser Basin is the most dynamic area of Yellowstone with the most varieties of hot springs, geysers, fumaroles, and mud volcanoes, and Norris has the highest measured temperature in Yellowstone and a high number of acidic hydrothermal features, including the world's largest known acidic geyser. Within this dynamic environment, there are unusual endolithic microbial communities living within the pore spaces of rocks that are excellent examples of potential biosignature preservation features to look for in hydrothermal remnants from early earth or other habitable worlds (Walker *et al.*, 2005).

Queen's Laundry is a region of the park that hosts a large spring mound where active silicification of microbes is being studied (Smythe *et al.*, 2016). Deep within the Hayden Valley lies the Obsidian Pool hot spring that hosts siliceous stromatolites that are another example of a key preservation feature possible within hydrothermal systems (Berelson *et al.*, 2011; Pepe-Ranney *et al.*, 2012).

### 3.2. Other analog sites presented at the workshop

Although Yellowstone is an interesting location to study habitability of extreme environments, it is not directly applicable to all Solar System bodies because the fluids present interact with a granitic crust, which, for example, has not been found to date on Mars (McSween, 2015; Filiberto, 2017).

To constrain the potential habitability of sites on other planetary bodies, it is vital that analog sites replicate crustal conditions (*e.g.*, temperature, pressures, and rock and fluid compositions) as close as possible as those they are an analogue for or account for said differences. Therefore, presentations at the meeting focused on other analog sites specifically to understand the habitability of early Mars. These sites included the following: fumaroles and hot springs at Rio Tinto as analogues for deposits explored in Gusev Crater by the Mars Exploration Rover Spirit (Ruff *et al.*, 2019), hydrothermal sediments and siliceous sinters at Tikitere Geothermal field in Taupo, New Zealand, as an analogue for the opaline silica deposits found at Columbia Hills, Gusev Crater, Mars (Dobson *et al.*, 2019), mafic magmas interacting with groundwater to produce a hydrothermal environment in the San Rafael Swell on the Colorado Plateau as applicable to environments on Early Mars (Filiberto *et al.*, 2019; Perl *et al.*, 2019; Costello *et al.*, 2020; Crandall *et al.*, 2021), and a closed basin paleolake at Pilot Valley Utah as an analogue for hypersaline fluvial and lacustrine deposits on Early Mars (Lynch, 2019).

To constrain the habitability of Gusev Crater and Early Mars, silica sinters and acid-sulfate leaching at both Rio Tinto and Tikitere Geothermal Field were investigated (Dobson *et al.*, 2019; Ruff *et al.*, 2019). The Mars Exploration Rover Spirit analyzed both sulfur-rich soils and deposits of opaline silica near the Home Plate deposit in Gusev Crater (Yen *et al.*, 2008; Ruff *et al.*, 2011). These are thought to represent fumarolic acid-sulfate leaching and a hot spring deposit, respectively (Ruff and Farmer, 2016). Constraints from these sites on Earth show that volcanic hydrothermal systems are both habitable environments, but may also have high preservation potential (Ruff and Farmer, 2016).

Similarly, Filiberto *et al.* (2019), and Perl *et al.* (2019) investigated a mafic dike that was hydrothermally altered from contact with groundwater as it was emplaced, expanding on previous work (Costello *et al.*, 2020). These results show that a high-temperature (>700°C) hydrothermal system was produced. Finally, Lynch (2019) investigated Pilot Valley in Utah, which is a closed basin paleolake that consists of hypersaline fluvial and lacustrine deposits. Specifically, the geochemistry and mineralogy at Pilot Valley are similar to multiple martian paleolake basins (Lynch *et al.*, 2015), and therefore, the habitable environment of Pilot Valley and potentially the microbiology present (Lynch *et al.*, 2019) could be used to constrain the habitability (and potential biology) of such ancient systems on Mars.

Earth is currently our only data point for life and by definition our best analogue for habitable environments; however, not all locations on Earth are relevant analogues to constrain habitability of other planets. Here, the focus was on habitability of extreme environments for understanding specifically the habitability of early Mars. These analog sites show that there could have been a range of habitable environments with the potential for some of these (especially silica-sinters) to preserve evidence of life.

### 3.3. Application to exoplanets

Understanding the environmental conditions (*e.g.*, atmospheric composition, stellar environment) that were present during the FBY on the prebiotic Earth provides constraints for identifying exoplanets with the potential for life. Similarly, understanding the conditions that define habitable environments and those that bracket environmental suitability, as well as the resulting local and global biosignatures, better enables us to search for habitable exoplanets.

The first confirmed exoplanets, or planets around other stars, were discovered around a neutron star in 1992 from the Arecibo Observatory in Puerto Rico (Wolszczan and Frail, 1992; Wolszczan, 1994). These so-called pulsar planets are rare and were probably formed from the leftover material after the formation of the neutron star (Wolszczan, 2012; Martin *et al.*, 2016). Since then, over 4000 exoplanets have been discovered (NASA Exoplanet Archives). Many thousands more are expected to be confirmed or detected by current space observatories such as NASA's Transiting Exoplanet Survey Satellite (Barclay *et al.*, 2018). Meanwhile, more planets are being detected by many ground observatories worldwide. Exoplanet science is moving from its first three decades of mostly planet detections, usually only knowing their orbits and sizes, toward exoplanet characterization, including atmospheric properties or even potential biosignatures (Fujii *et al.*, 2018; Madhusudhan, 2019).

Most stars have one or more planets, from small Earth-sized to big Jupiter-sized worlds (Cassan *et al.*, 2012). Only those of spectral types F, G (like our Sun), K, and M live long enough for life to form, evolve, and thrive on their planets, assuming this process takes about 1 billion years (Safonova *et al.*, 2016). The smaller red dwarf stars (M type) are the most common (75%), live longer, and are also more likely to hold smaller Earth-sized planets than Sun-like stars (Toumi *et al.*, 2019).

Unfortunately, at star-planet distances that allow for surface liquid water, planets around red dwarf stars are also prone to be tidally locked (one face always pointing toward the star) and experience large stellar radiation fluctuations, especially during their early stages, which might erode or strip their atmosphere altogether (Shields *et al.*, 2018). Also, red dwarf stars might not emit enough UV radiation to start the photochemistry and prebiotic synthesis necessary for the development of life (Rimmer *et al.*, 2018). Thus, there is extensive debate about the potential habitability of red dwarf stars.

About 30% of the stars have a potentially habitable planet (*i.e.*, an Earth-sized planet in the habitable zone), some (2%) even have more than one planet in the habitable zone (Zink and Hansen, 2019). These planets are less frequent around sun-like stars (20%) and are more frequent around red dwarf stars (50%) (Petigura *et al.*, 2013; Kopparapu, 2013; Dressing and Charbonneau, 2015; Zink and Hansen, 2019). So far, there are up to 60 potentially habitable planets detected (PHL Habitable Exoplanet Catalog), most around red dwarf stars. They are further divided into 24 Earth-sized (*i.e.*, radius ≤1.5 Earth radii or mass ≤5 Earth masses) and 36 Super-Earths (*i.e.*, radius >1.5 Earth radii or mass >5 Earth masses). Earth-sized planets are more likely rocky in composition, but Super-Earths include the transition from rocky, to ocean, to minigas planets (*i.e.*, mini-Neptunes).

The habitability of rocky Super-Earths is a topic of controversy regarding whether they would be in a state of Earth-style plate tectonics, stagnant lid, or some unknown tectonic state, with plate recycling possibly being required for life as we know it (O'Neill and Lenardic, 2007; Valencia *et al.*, 2007; Korenaga, 2010). The TRAPPIST-1 system is the best-known exoplanet system and an excellent laboratory for habitability studies of planets around red dwarf stars (Gillon *et al.*, 2017). Up to three or four of its seven Earth-sized planets are considered potentially habitable.

Future missions such as the James Webb Space Telescope (JWST) might have the capability to observe the atmospheres of the planets around TRAPPIST-1 (Lustig-Yaeger *et al.*, 2019). JWST should be able to determine if planets around red dwarf stars can hold habitable environments by constraining the atmospheric water content. Most of the known potentially habitable planets are older than Earth or their ages are unknown. Future space missions such as European Space Agency's PLAnetary Transits and Oscillations of stars (PLATO) should provide more information about their ages (Rauer *et al.*, 2013). Therefore, in the next decade we should have information about the atmospheric composition of many nearby planets of different ages (<50 light-years away). These objects will provide an excellent opportunity to compare with Earth to understand atmospheric evolution and habitability of planets from their FBY to the present (Krissansen-Totton *et al.*, 2018).

### 3.4. Open questions

From the research presented at the conference, as well as the discussion periods, several key open questions were identified. Although these questions are not necessarily centered on the concept of “the first billion years,” they are motivated by this context and follow the conference's three themes.

#### 3.4.1. Origins of life

The ultimate context to furthering the search for life beyond Earth is an understanding of how life arose on it. Although much work has been done since the early origins of life experiments, the details of abiogenesis are still unknown. Thus, the overarching fundamental question remains,

How, when, and where did life begin on Earth?

By better characterizing the chemical pathways for abiogenesis and the environmental context in which it took place, we can also begin to refine our search for habitable bodies beyond Earth. For example, we could begin to answer,

What are the mechanisms that could lead to abiogenesis on ocean worlds?

In our Solar System alone, there are several icy moons that with certainty are ocean worlds: Europa, Ganymede, Enceladus, and Titan (Carr *et al.*, 1998; Iess *et al.*, 2012, 2014; Saur *et al.*, 2015). Other icy worlds may also harbor oceans beneath thick icy crusts, for example, Callisto, Dione, Triton, and Pluto (Carr *et al.*, 1998; Hussmann *et al.*, 2006; Zannoni *et al.*, 2020). As we continue to study these worlds, refining our knowledge of Earth's FBY can help guide Solar System exploration.

#### 3.4.2. Habitability

Once life has emerged on a body, appropriate conditions are required to enable its establishment and propagation over time. Understanding the conditions that were present during the establishment of life on early Earth, as well as the conditions on Solar System bodies, allows us to gain insights into the conditions that may be required for life. Thus, it is important to understand,

What habitable environments on early Earth are applicable to other planetary bodies?

Terrestrial extremophiles have evolved to tolerate a large range of environmental conditions (*e.g.*, pH-level, temperature, salinity, radiation, pressure, concentration of toxic metals), but there are some environmental stressors that life cannot tolerate and modern conditions may not fully reflect the environments that emerging life experienced on the early Earth (Rothschild and Mancinelli, 2001; National Research Council, 2007; Conrad, 2014; Cockell *et al.*, 2016; National Academies of Sciences, Engineering, and Medicine, 2019).

Specifically, environmental conditions on Early Earth were likely more extreme than today; such conditions may be more applicable to other planetary bodies (National Research Council, 2007). Constraining the conditions on early Earth (*e.g.*, volatile budget and specifically the carbon inventory) would thus help to decipher the biologic potential of other bodies. Searching for relics of prebiotic chemistry on Earth, and other planetary bodies, may also help constrain the environmental parameters in place for emerging life. Furthermore, studying Solar System bodies where life may still be emerging (*e.g.*, Titan) may help provide insight to potential habitable conditions on early Earth (National Academies of Sciences, Engineering, and Medicine, 2019). Building on environmental habitable conditions, fundamental questions remain such as,

What are the limiting environmental conditions (*e.g.*, temperature, pressure, fluid composition, radiation) that permit life to metabolize and replicate (*i.e.*, what environments are too extreme)?

The FBY of Solar System history were traumatic as the remains of planet formation impacted the young bodies. Although much debate remains on the topic, a so-called Late Heavy Bombardment (LHB) is thought to have occurred around 3.9 Ga (Tera *et al.*, 1974; Gomes *et al.*, 2005; Levison *et al.*, 2011; Bottke and Norman, 2017). Such events motivate the question,

How does impact cratering affect the origin and establishment of life?

Although early impacts may have been energetic enough to vaporize seas (Thomas *et al.*, 1997), modeling has shown that the LHB would not have fully sterilized the early Earth (Abramov and Mojzsis, 2009). Indeed, impacts may have even facilitated prebiotic chemistry by forming subsurface hydrothermal systems (Sleep and Zahnle, 1998; Kring, 2004, 2019).

#### 3.4.3. Search for life

Analog research studies provide the important biogeochemical context to how terrestrial organisms interact with their environment. These studies provide biosignatures that improve our search for life beyond Earth. For example, chemical and isotopic signatures in stromatolites and other rocks have been interpreted to be a result of biological activity (Schidlowski, 1988). Further studies are needed with a mission perspective to answer,

What biosignatures could be used by *in situ* instrumentation on rovers/landers to confidently assess ancient extraterrestrial environments?

Furthermore, with the now over 4000 discovered exoplanets, a global view on biosignatures is needed to refine our understanding of,

How do we detect habitable and potentially inhabited exoplanets?

With upcoming advanced exoplanet search missions (*e.g.*, JWST), it becomes imperative for exoplanet astronomers, mission engineers, and biosignature researchers to work synergistically so that we can further our understanding of the potential for life in the Universe. However, life on other planets may be different than life as we know it; thus,

How do we expand the definition of habitability and life to include these potential differences?

Terrestrial life can help guide the search for some aspects of life in the Universe; however, life may organize itself in a different manner on other planetary, even from those in the most extreme terrestrial environments (National Research Council, 2007; Johnson *et al.*, 2018; National Academies of Sciences, Engineering, and Medicine, 2019). Therefore, research into agnostic biosignatures, which specifically does not presuppose any particular molecular framework, is needed to improve our chances of finding the potentially diverse manifestation of life (Johnson *et al.*, 2018).

## 4. Conclusions

The context of Earth's and the Solar System's FBY provides vital clues to understanding the emergence and establishment of life. The large-scale processes that set up the geochemical context (*e.g.*, accretion, differentiation) and acted globally over time (*e.g.*, asteroidal and cometary bombardment) allow us to understand the potential chemistry that eventually led to life on Earth, as well as the environment that allowed for its propagation. Such research, especially through the lens of terrestrial analog studies, allows us to refine Solar System exploration strategies as we search for life beyond Earth. The FBY Habitability conference was intended to provide the venue that would facilitate such multidisciplinary discussions. This Special Collection presents both research and review articles that resulted from the conference.

## Abbreviations Used

αHAs: α-hydroxy acids
FBY: first billion years
GOE: Great Oxidation Event
JWST: James Webb Space Telescope
LHB: Late Heavy Bombardment
UV: ultraviolet
